# Impact of visceral adiposity on severity of acute pancreatitis: a propensity score-matched analysis

**DOI:** 10.1186/s12876-019-1015-z

**Published:** 2019-06-13

**Authors:** Jiarong Xie, Lu Xu, Yuning Pan, Peifei Li, Yi Liu, Yue Pan, Lei Xu

**Affiliations:** 1 College of Medicine, Ningbo University, Fenghua Road, Jiangbei District, Ningbo, Zhejiang Province, 818 NO China; 2 Department of Gastroenterology, Ningbo First Hospital, No.59 Liuting Street, Haishu District, Ningbo, Zhejiang Province, China; 3 Department of Radiology, Ningbo First Hospital, No.59 Liuting Street, Haishu District, Ningbo, Zhejiang Province, China; 4 Laboratory of Digestive Diseases, Ningbo First Hospital, No.59 Liuting Street, Haishu District, Ningbo, Zhejiang Province, China

**Keywords:** Visceral adiposity, Skeletal muscle tissue, Acute pancreatitis, Predictor, Computed tomography

## Abstract

**Background:**

The relationship between visceral adiposity and acute pancreatitis (AP) has not been completely elucidated. This study evaluated the significance of visceral adipose tissue (VAT) and the ratio of VAT to skeletal muscle tissue (VAT/SMT) in the prognosis of AP patients.

**Methods:**

Based on a 1:2 propensity score matching, 306 hospitalized patients were enrolled in the study analysis from 2010 to 2017. VAT, subcutaneous adipose tissue (SAT), and SMT were measured using unenhanced computed tomography (CT). Cox proportional hazards models were applied for the analysis.

**Results:**

VAT and the VAT/SMT ratio were significantly higher in the severe AP (SAP) and moderately severe AP (MSAP) groups compared to the mild AP (MAP) group (both *p* < 0.001). Intensive care transfer, AP severity, systemic complications, and prognostic scores (Acute Physiology and Chronic Health Evaluation II [APACHE-II] score ≥ 8, Ranson’s score ≥ 3, Bedside Index of Severity in Acute Pancreatitis [BISAP] score ≥ 3, and the systemic inflammatory response syndrome [SIRS] score ≥ 2) significantly correlated with VAT and the VAT/SMT ratio in AP patients. The multivariate adjusted hazard ratios (HRs) for VAT and the VAT/SMT ratio in the relationship of body parameters and AP mortality were 1.042 (95% confidence interval (CI), 1.019–1.066) and 7.820 (95% CI, 1.978–30.917), respectively. Compared with other prognostic scores, VAT had the highest area under the curve of receiver operating characteristics (ROC) (0.943, 95% CI, 0.909–0.976).

**Conclusion:**

High VAT and VAT/SMT ratio are independent negative prognostic indicators of AP.

**Trial registration:**

Clinical study registration number: NCT03482921. Date of registration: 03/23/2018.

## Background

Acute pancreatitis (AP) is cause by inflammation of the pancreas. In the majority of cases, AP is mild and self-limited. However, about 20% of AP patients experience severe complications with a high risk of mortality [1, 2]. It is vital to classify severe AP cases early in the evolution of the disease, because severe cases require more aggressive treatment to achieve the best possible results and reduce complications [3]. Historically, several scoring systems have been developed and widely utilized to determine AP severity: Acute Physiology and Chronic Health Evaluation II (APACHE-II) score, Bedside Index of Severity in Acute Pancreatitis (BISAP) score, and Ranson’s score [4–6]. Each of these scoring systems has its advantages and limitations. Other systems have also been developed and many others are currently being created. However, it has remained difficult to identify the most effective method to predict AP severity at an early stage.

It is well-established that obesity (defined as body mass index (BMI) > 30 kg/m^2^ by the World Health Organization) is a risk factor for AP severity and increases the incidence of systemic complications and mortality [7–9]. BMI measurements and waist circumference are often viewed as indicators of being overweight. One disadvantage of these parameters is that they do not distinguish between truncal obesity and visceral obesity. This is significant because fat distribution plays an important role in AP severity. In recent years, many parameters related to sarcopenic obesity, including visceral adipose tissue (VAT), subcutaneous adipose tissue (SAT), skeletal muscle tissue (SMT), and the VAT/SMT ratio, have been considered important risk factors of AP [10–13]. Some recent reports indicated that increased VAT is more closely associated with AP severity compared to BMI [10, 14–16]. However, it is unclear which parameter is the best predictor of AP severity [17]. There are very few studies in the literature that address the association between body composition parameters and clinical outcomes in AP. Thus, this study explored the association between these parameters and AP severity to elucidate which is the most suitable predictor of AP.

## Methods

### Study population

**Fig. 1:**
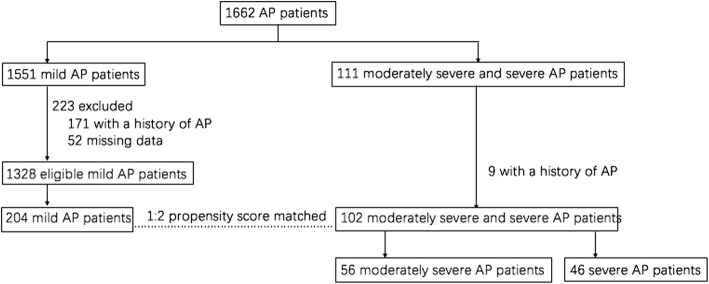
Study flowchart

We analyzed 1662 consecutive hospitalized patients with AP between August 1st, 2010 and August 31st, 2017 at Ningbo First Hospital, China. This study was approved by the Human Ethics Committee. The clinical study registration number is NCT03482921. Severe AP (SAP) and moderately severe AP (MSAP) were defined in 111 patients according to the Revised Atlanta Classification of AP [18] (Fig. 1). Exclusion criteria for the study included age < 18 years, missing data in the electronic medical record, and history of AP. Nine unqualified patients were excluded. SPSS R plug-in (SPSS R Essentials) was applied for matching [19]. We used the SPSS “PS Matching” feature to perform propensity score-matched analysis. Matching factors include age, sex, and BMI. SAP, and MSAP patients were matched 1:2 in a multivariable logistic analysis using stepwise regression based on a greedy matching algorithm with a caliper of 0.2 times the standard deviation (SD) of the logit. After applying 1:2 propensity score matching, 102 eligible patients were matched to 204 patients with mild AP (MAP). In total, 306 hospitalized patients were enrolled in this study.

### Severity assessment

AP severity was defined using the Revised Atlanta Classification criteria. The clinical classification defined 3 grades of severity: MAP, MSAP, and SAP.

### Definition of systemic complications

Systemic complication was defined as a score ≥ 2 in one of three assessed organ systems (respiratory, cardiovascular, or renal) according to the modified Marshall scoring system [20].

### Definition of local complications

Local complications of AP were divided into: acute peri-pancreatic fluid collection (APFC), acute necrotic collection (ANC), pancreatic pseudocyst, and walled-off necrosis (WON). Diagnosis of local complication on unenhanced computed tomography (CT) was evaluated by two experienced radiologists who were blinded to detailed information about the cases. When necessary, follow-up contrast-enhanced CT (CECT) and magnetic resonance imaging (MRI) are performed based on the clinical status of patients, including persistence or recurrence of abdominal pain, increasing organ dysfunction, and development of clinical signs of sepsis. Once patients are diagnosed with APFC, ANC, WON, or pseudocyst in the follow-up imaging examinations, they are considered to present with local complications.

### Laboratory tests

Creatinine, calcium, and albumin levels obtained within the first 24 h were collected for each patient. Additional variables included intensive care unit (ICU) requirement, length of hospital stay, and in-hospital mortality.

### Combined scoring systems

The APACHE-II, BISAP, and SIRS scores were calculated within the first 24 h of admission, and Ranson’s score was recorded within 48 h of admission.

### CT and image analysis

**Fig. 2:**
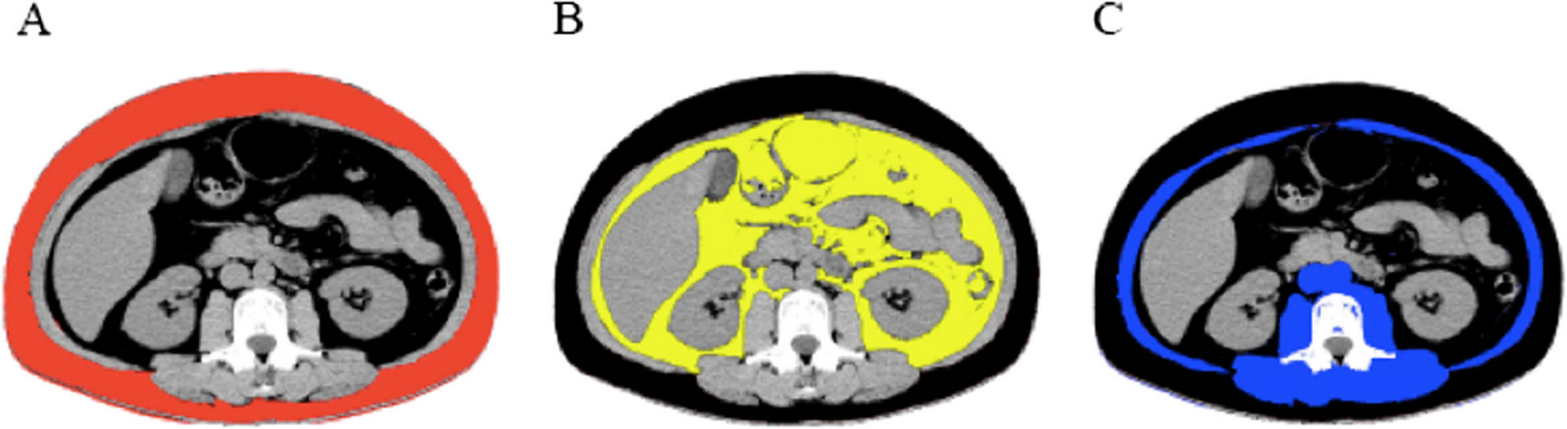
**a** Subcutaneous adipose tissue (SAT) (red) was calculated within the region of interest (ROI) by selection of pixels at the L3/4 intervertebral space **b** Visceral adipose tissue (VAT) (yellow) **c** Skeletal muscle tissue (SMT) (blue)

A dual 16-slice CT scanner (SIMENS SOMATOM Sensation; Siemens, Germany) was used for entire / upper abdominal CT at 250 mA; tube voltage, 120 kV; data collection thickness 5 mm; reconstruction thickness 1 mm; and reconstruction interval 1 mm. CT scans were performed within 24 h after patient admission. Entire abdomen CT was performed to 165 patients. Upper abdomen CT was performed to 141 patients. Two consecutive, axial images on unenhanced CT at the L3/4 intervertebral space were retrospectively reviewed by two radiologists who were blinded to patient information. We calculated fat and muscle cross-sectional areas, which have been validated as the best proxies of SAT and VAT independent of age [21]. Skeletal muscle areas, including the psoas, paraspinal, and the abdominal wall muscles excluding the intra-abdominal visceral muscles, were also measured (Fig. 2). We used the Photoshop “magic wand” to outline and measure regions of similar signal intensity based on pixel counts. We manually outlined the margin of fat deposition manually in fat-tissue regions with poor contrast, which may be a more accurate way to measure compartment volumes. The Hounsfield units (HU) range for adipose tissue was − 190 to − 30 HU [22]. The mean value of the two images was calculated. The abdominal muscular wall distinguishing between VAT and SAT was traced automatically and adjusted manually [23, 24]. The VAT/SMT ratio was calculated by dividing VAT by SMT. All CT scans were analyzed using a commercially available software program (Photoshop CS6, Adobe Systems, CA) [25].

### Statistical analysis

Continuous variables are expressed as mean ± standard deviation (SD) and median (interquartile range). Categorical outcomes are presented as frequencies and proportions. Comparisons of different variables were performed using one-way analysis of variance (ANOVA) and Likelihood Ratio. Cox regression analysis adjusted for confounders were used to identify the relationships. Discrimination of the predicting factors, including VAT and VAT/SMT ratio, were accomplished via receiver operating characteristic (ROC) curves. A two-tailed *p* value < 0.05 was considered statistically significant. All statistical analyses were performed using SPSS software (version 23.0; SPSS, Chicago, IL).

## Results

### Baseline characteristics

In this study, every MSAP and SAP patient was propensity score matched with another MAP patient at a ratio of 1:2. Finally, 306 hospitalized patients were enrolled for further analysis. Among the 306 patients, causative factors of AP included gallstone-related (142; 46.4%), idiopathic (91; 29.7%), hypertriglyceridemia (48; 15.7%), alcohol abuse (22; 7.2%), and others (3;1.0%). The mean age of the patients was 50.6 ± 18.7 years. The mean estimated BMI was 24.4 ± 4.5 kg/m^2^. The mean SAT value was 131.1 ± 23.1 cm^2^. The mean VAT value was 136.1 ± 27.3 cm^2^. The mean SMT value was 138.2 ± 25.8 cm^2^.

### Comparisons of characteristics according to AP severity

Age, sex, and BMI distribution were not statistically different among groups after propensity matching. There was a significant association between AP severity and individual fat parameters, except for SAT (Table 1). VAT and the VAT/SMT ratio were significantly higher in the MSAP and SAP groups compared to the MAP group (both *p* < 0.001). The mean SMT values decreased as AP severity increased (p < 0.001).

**Table 1:** Clinical characteristics of patients stratified by AP severity

	Total	Severity of AP			*p* value
		Mild	Moderately severe	Severe	
	(*n* = 306)	(*n* = 204)	(*n* = 56)	(*n* = 46)	
Age (years)	50.6 ± 18.7	50.4 ± 18.7	49.4 ± 18.9	52.8 ± 19.3	0.632
Sex, male (%)	157 (51.3%)	107 (52.5%)	25 (44.6%)	25 (54.3%)	0.552
BMI (kg/m^2^)	24.4 ± 4.5	24.2 ± 5.0	24.7 ± 2.7	25.3 ± 3.1	0.505
Fat volume parameters (cm^2^)					
SAT	131.1 ± 23.1	128.1 ± 22.1	138.8 ± 22.4	134.6 ± 25.8	0.04
VAT	136.1 ± 27.3	121.6 ± 17.3	154.1 ± 15.1	178.7 ± 15.2	< 0.001
SMT	138.2 ± 25.8	143.5 ± 25.8	130.6 ± 20.2	123.7 ± 24.5	< 0.001
VAT/SMT ratio	1.03 ± 0.32	0.87 ± 0.21	1.21 ± 0.21	1.49 ± 0.26	< 0.001

*BMI*, Body mass index; *SAT*, Subcutaneous adipose tissue; *VAT*, Visceral adipose tissue; *SMT*, Skeletal muscle tissue

Student Newman-Keuls post hoc comparisons of fat volume parameters were assessed between three groups (*p* < 0.05, respectively)

### Relationship between body parameters and severity outcomes

Based on the VAT value at the L3/4 intervertebral space, patients were separated into five groups. ICU transfer, AP severity, and systemic complications (APACHE-II scores ≥8, BISAP score ≥ 3, SIRS score ≥ 2) were significantly related to VAT values in the AP patients (Table 2).

**Table 2:** Relationships between VAT and severity outcomes

VAT(cm^2^)	< 90	90–120	120–150	150–180	> 180	*p* value
Number of patients	7	97	103	69	30	
ICU transfer	0 (0%)	2 (2.1%)	5 (4.9%)	27 (39.1%)	24 (80.0%)	< 0.001
SAP	0 (0%)	0 (0%)	4 (3.9%)	13 (18.8%)	29 (96.7%)	< 0.001
Mortality	0 (0%)	0 (0%)	4 (3.9%)	8 (11.6%)	15 (50%)	< 0.001
Systemic complications	0 (0%)	3 (3.1%)	10 (9.7%)	40 (58.0%)	29 (96.7%)	< 0.001
Local complications						
APFC	2 (28.6%)	38 (39.2%)	28 (27.2%)	26 (37.7%)	16 (53.3%)	0.107
ANC	0 (0%)	0 (0%)	0 (0%)	8 (11.6%)	3 (10.0%)	< 0.001
WON	0 (0%)	1 (1.0%)	5 (4.9%)	6 (8.7%)	4 (13.3%)	0.038
Pancreatic pseudocyst	0 (0%)	2 (2.1%)	6 (5.8%)	8 (11.6%)	3 (10.0%)	0.088
Prognostic scores						
APACHE-II score ≥ 8	0 (0%)	7 (7.2%)	8 (7.8%)	17 (24.6%)	21 (70.0%)	< 0.001
Ranson’s score ≥ 3	0 (0%)	7 (7.2%)	6 (5.8%)	9 (13.0%)	14 (46.7%)	< 0.001
BISAP score ≥ 3	0 (0%)	2 (2.1%)	3 (2.9%)	4 (5.8%)	5 (16.7%)	0.048
SIRS score ≥ 2	1 (14.3%)	12 (12.4%)	22 (21.4%)	40 (58.0%)	21 (70.0%)	< 0.001

*VAT*, Visceral adipose tissue; *ICU*, Intensive care unit; *SAP*, Severe acute pancreatitis; *APFC*, Acute peripancreatic fluid collection; *ANC*, Acute necrotic collection; *WON*, Walled-off necrosis; *APACHE-II*, Acute Physiology and Chronic Health Evaluation II; *BISAP*, Bedside Index of Severity in Acute Pancreatitis; *SIRS*, systemic inflammatory response syndrome

Patients were also separated into five groups according to the VAT/SMT ratio. ICU transfer, AP severity, systemic complications, and prognostic scores (APACHE-II scores ≥8, Ranson’s score ≥ 3, BISAP score ≥ 3, SIRS score ≥ 2) had significant relationship with the VAT/SMT ratio (Table 3).

**Table 3:** Relationship between the VAT/SMT ratio and severity outcomes

VAT/SMT ratio	< 0.6	0.6–0.9	0.9–1.2	1.2–1.5	> 1.5	*p* value
Number of patients	7	147	60	56	36	
ICU transfer	0 (0%)	5 (3.4%)	6 (10.0%)	22 (39.3%)	25 (69.4%)	< 0.001
SAP	0 (0%)	2 (1.4%)	5 (8.3%)	11 (19.6%)	28 (77.8%)	< 0.001
Mortality	0 (0%)	2 (1.4%)	4 (6.7%)	8 (14.3%)	13 (36.1%)	< 0.001
Systemic complications	0 (0%)	7 (4.8%)	19 (31.7%)	28 (50%)	28 (77.8%)	< 0.001
Local complications						
APFC	2 (28.6%)	49 (33.3%)	22 (36.7%)	22 (39.3%)	15 (41.7%)	0.859
ANC	0 (0%)	0 (0%)	4 (6.7%)	5 (8.9%)	2 (5.6%)	0.002
WON	0 (0%)	2 (1.4%)	5 (8.3%)	4 (7.1%)	5 (13.9%)	0.017
Pancreatic pseudocyst	0 (0%)	2 (1.4%)	3 (5.0%)	9 (16.1%)	5 (13.9%)	0.01
Prognostic scores						
APACHE-II score ≥ 8	0 (0%)	13 (8.8%)	8 (13.3%)	13 (23.2%)	19 (52.8%)	< 0.001
Ranson’s score ≥ 3	0 (0%)	9 (6.1%)	6 (10.0%)	8 (14.3%)	13 (36.1%)	< 0.001
BISAP score ≥ 3	0 (0%)	2 (1.4%)	1 (1.7%)	5 (8.9%)	6 (16.7%)	0.002
SIRS score ≥ 2	1 (14.3%)	22 (15.0%)	17 (28.3%)	32 (57.1%)	24 (66.7%)	< 0.001

*VAT*, Visceral adipose tissue; *SMT*, Skeletal muscle tissue; *ICU*, Intensive care unit; *SAP*, Severe acute pancreatitis; *APFC*, Acute peripancreatic fluid collection; *ANC*, Acute necrotic collection; *WON*, Walled-off necrosis; *APACHE-II*, Acute Physiology and Chronic Health Evaluation II; *BISAP*, Bedside Index of Severity in Acute Pancreatitis; *SIRS*, Systemic inflammatory response syndrome

### Confounding variables

Further analyses were conducted for creatinine, calcium, and albumin levels using an adjusted model (Table 4). The multivariate adjusted hazard ratios (HRs) for VAT and the VAT/SMT ratio in the relationship between body parameters and AP mortality were 1.042 (95% confidence interval (CI), 1.019–1.066) and 7.820 (95% CI, 1.978–30.917), respectively. The VAT/SMT ratio was positively related to the incidence of AP mortality (*p* < 0.001).

**Table 4:** Multivariate adjusted HRs and 95% CIs for the association between body parameters and AP mortality

Variables	All of the participants(*n* = 264)			
	Model1		Model2	
	HR (95% CI)	*p* value	HR (95% CI)	*p* value
VAT, cm^2^	1.042 (1.019–1.066)	< 0.001		
VAT/SMT ratio			7.820 (1.978–30.917)	0.003
Creatinine	1.003 (1.001–1.006)	0.014	1.004 (1.001–1.007)	0.005
Calcium	0.498 (0.103–2.413)	0.387	0.232 (0.045–1.184)	0.079
Albumin	0.986 (0.921–1.056)	0.682	0.992 (0.925–1.063)	0.812

VAT, visceral adipose tissue; SMT, skeletal muscle tissue

Model 1 and model 2 were based on the VAT and the VAT/SMT ratio, respectively. Cox proportional hazard models were used to estimate the HRs, 95% Cis, and *p* values

### Comparisons of independent predictors

**Fig. 3:**
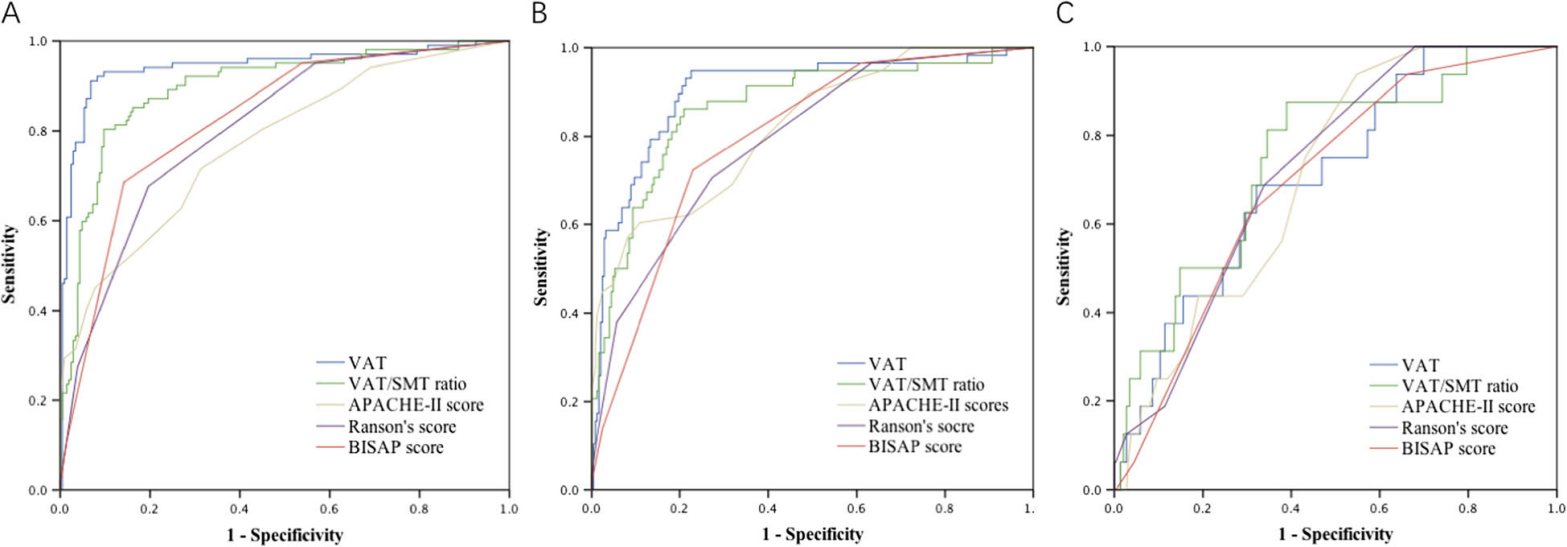
ROC analysis. **a** Diagnostic assessment of the independent predictors for acute pancreatitis (AP) severity; **b** Diagnostic assessment of the independent predictors for the incidence of intensive care unit (ICU) stay; **c** Diagnostic assessment of the independent predictors for the incidence of walled-off necrosis (WON)

The AUCs were measured to evaluate individual fat parameters of VAT and the VAT/SMT ratio for predicting the incidence of SAP and MSAP, ICU stay, and the incidence of WON (Fig. 3).

VAT had the highest area under the curve of ROC (0.943, 95% CI, 0.909–0.976) for predicting the incidence of SAP and MSAP, among independent predictors. The AUC of the VAT/SMT ratio was also significantly higher compared to the predictive scoring systems (0.896, 95% CI, 0.856–0.936). The optimal cut-off value of VAT for the prognosis of AP was 145.0. The sensitivity and specificity of this cut-off value were 90.2 and 93.1%, respectively. The optimal cut-off point of the VAT/SMT ratio for the prognosis of AP was 0.706. The sensitivity and specificity of this cut-off value were 80.4 and 90.2%, respectively. The cut-off values for VAT and the VAT/SMT ratio can be used to predict the incidence of SAP and MSAP.

We also demonstrated that VAT and the VAT/SMT ratio had high sensitivity and specificity for predicting the incidence of ICU stay and WON compared to other predictive scoring systems. The AUC of VAT for predicting the incidence of ICU stay was 0.900 (95% CI, 0.851–0.949). The second highest AUC of ROC was the VAT/SMT ratio (0.865, 95% CI, 0.810–0.921). The AUC of the VAT/SMT ratio was the highest predictor of the incidence WON (0.745, 95% CI, 0.628–0.863).

## Discussion

This propensity score-matched, case-control study assessed the significance of VAT and the VAT/SMT ratio as AP prognostic factors. VAT and the VAT/SMT ratio increased as AP severity increased, including the incidence of SAP and MSAP, the incidence of ICU transfer, and the incidence of WON. This study also calculated the optimal cut-off values, which can be used to predict AP severity.

AP involves sudden inflammation in the pancreas. Early prediction of SAP will allow more aggressive management at early stages of the disease. There are many SAP predictive scoring systems. However, none can predict the severity at early stages of AP. If initial CT scans in AP patients are routinely performed at an early stage, the values of VAT and the VAT/SMT ratio could be quickly calculated and used as predictors.

Obesity is a well-established risk factor of severity and mortality in AP patients [9, 26]. In previous studies, obesity was determined as BMI > 30 kg/m^2^. However, Asians tend to have a significantly lower BMI, at approximately 2–3 kg/m^2^ lower compared to age and sex matched whites with the same amount of body fat [27]. Currently, there is no universal tool to predict prognosis and mortality in AP patients [28–33]. In addition, the scoring tools do not distinguish between truncal obesity and visceral obesity. Some studies showed that VAT significantly correlates with poor outcomes in AP patients [10, 14–16]. Therefore, we investigated the relationship between body parameters, prognosis, and mortality in AP patients. This study showed that VAT and the VAT/SMT ratio were significantly higher in the MSAP and SAP groups compared to the MAP group. Besides, VAT, and VAT/SMT ratio had the highest AUC in predicting the incidence of SAP and MSAP, the incidence of ICU transfer, and the incidence of WON. VAT and the VAT/SMT ration could therefore be used to distinguish AP severity by applying VAT and the VAT/SMT ratio scoring in clinical practice.

Previous studies reported gender-based variations in fat distribution, especially in obese patients [34, 35]. Risk of disease, as well as visceral fat, increases dramatically with age [36, 37]. In our study, every high-risk case was propensity score matched at a 1:2 ratio with another patient with MAP. Matching was based on patient demographics (age, sex, and BMI), which decreased bias and effectively controlled for confounding variables. Some clinical characteristics, such as creatinine, calcium, and albumin levels, are typically considered biomarkers associated with disease prognosis and mortality. These characteristics have often been considered in the AP scoring systems [4, 38, 39]. We used Cox proportional hazard models adjusted for creatinine, calcium, and albumin levels, which may reduce the bias. The mean value of two images were reviewed by two dedicated radiologists blinded to clinical and demographic data to increase the accuracy of our results.

There are four studies that have presented similar results to the finding described here. A study of 62 cases [15] reported a close relationship between AP severity, systemic complications, and individual fat parameters. Another study of 124 patients [14] demonstrated that VAT volume is significantly associated with SAP but not BMI or waist circumference. Yoon et al. [10] found that high visceral fat with low skeletal muscle volume plays an important role in AP severity. Natu et al. [16] found that VAT is closely associated with organ failure and necrosis in AP. However, there are some limitations. Some of these studies did not focus on body parameter classifications. Furthermore, they did not provide clinical characteristics of the patients, such as creatinine, calcium, and albumin, which could act as confounding variables. Moreover, since the first two studies were published before the revised version appeared, they used the previous version of the Atlanta classification, which is not as precise as other measures. Our study investigated the relationship between different body parameters and AP severity. To our knowledge, this study is the largest describing these parameters.

There are several limitations in our study. First, at present, it is difficult to perform a CT scan on a patient within 24 h after admission in some countries and regions. Second, due to its retrospective design, the statistical power of analysis is relatively weak. Third, individuals from the Chinese population were included in our study. We did not recruit AP patients in western countries, which may increase potential bias.

This is the largest study exploring the relationship between different body parameters and severity of AP after taking several covariates into account. Furthermore, we calculated the optimal cut-off value of VAT and the VAT/SMT ratio. Therefore, VAT and the VAT/SMT ratio should be considered as independent predictors in AP patients. To extend our results to clinical-decision making, further randomized, multi-center, prospective studies that include different countries and regions are needed to better assess the association between visceral adiposity with severity of AP.

## Conclusions

The results of this study suggest that VAT and the VAT/SMT ratio are strong predictors of severity, mortality, and systemic complications in AP patients.

## Data Availability

The datasets generated in the current study are available from the corresponding author on reasonable request.

## Abbreviations

ANC: Acute necrotic collection
AP: Acute pancreatitis
APACHE-II: Acute Physiology and Chronic Health Evaluation II
APFC: Acute peripancreatic fluid collection
BISAP: Bedside Index of Severity in Acute Pancreatitis
BMI: Body mass index
CT: Computed tomography
ICU: Intensive care unit
MAP: Mild acute pancreatitis
MRI: Magnetic resonance imaging
ROC: Receiver operating characteristic
SAP: Severe acute pancreatitis
SIRS: Systemic inflammatory response syndrome
SMT: Skeletal muscle tissue
VAT: Visceral adipose tissue
WON: Walled-off necrosis
